# Supplementary material for: Chromothripsis during telomere crisis is independent of NHEJ, and consistent with a replicative origin
Source: Genome Res. 2019 May;29(5):737–49. doi: 10.1101/gr.240705.118 (PMC6499312; doi:10.1101/gr.240705.118)
Supplement: Supplemental Material [file supp_gr.240705.118_Supplemental_file_1.zip › contigs/annotated_contigs/DB38/contig.3.DB38_length_640_mean_cov_10.353125.docx]

**DB38_length_640_mean_cov_10.353125**

```
ATCTAATTAAAAGGGAAAACTCTCTATTTCAGGTTTATATGTATCAATTGCTTGATTAGAAAATAAATTATTTAACCCATATAATCTGG
 >chr1:142964758-142965172 + E=2e-222
CGTCAAAAAACTTATAATAAAATTACTTTGATTTTATTGCTTATAAGTTTGTGAATTTAAATCTCAACTGACAGTGATGAATTTATTTG

CTTTGTGCTGACAAGACAGTATGTTGAAGTTTTGTTTTCCAACTCAGTTCTAAATCTGCAGTGTATCAAAATCTCCTCATTATGCTTTG

GATGTTATGGTACATAAATGGTATCTTCACAAAGATATATTCATTCTGGTACTGAAAGTGCATACTTAAAATTCTTCATGAACAGTTTT

ATTGAAAAAATATGTAGACAAGCCATTAATTATACACAGTGATCTGGAACAAATTCTT|TTTTTTTT|TATAGCTAAAGGATTTATTTA
                                                                   >chr2:11929914-11930
TTCACTGAAGAAGAAATCCACATGGGCTCTGAAACTTAAAAGACATTAGTCCTTGTGATATGCATGCTAAATCTTTCTACAAG|ATTTA
018 + E=8e-51
T|ATTATAGCCACCAGAATATAGTCACAATCACTACCCAATTTTGATAACTGAAGATCATTTTTTGATTAATTGCATTGTTCAGGCTAT
  >chr4:162053142-162053250 - E=3e-53
CTTATCTTTTAGAAATATTTC
```
